# Spanish Translation and Cultural Adaptation of the Fibromyalgia Knowledge Questionnaire

**DOI:** 10.3390/ijerph18147678

**Published:** 2021-07-19

**Authors:** María Mendoza-Muñoz, Miguel Rodal, Miguel Ángel García-Gordillo, Ángel Acevedo-Duque, Judith García-Matador, José Ignacio Calzada-Rodríguez, Jesús Morenas-Martín

**Affiliations:** 1 Health, Economy, Motricity and Education Research Group (HEME), Faculty of Sport Sciences, University of Extremadura, 10003 Cáceres, Spain; mamendozam@unex.es (M.M.-M.); judgm@hotmail.com (J.G.-M.); jocalzada@alumnos.unex.es (J.I.C.-R.); 2 BioErgon Research Group, Faculty of Sport Sciences, University of Extremadura, 10003 Cáceres, Spain; 3 Universidad Autónoma de Chile, Talca 3467987, Chile; miguel.garcia@uautonoma.cl (M.Á.G.-G.); angel.acevedo@uautonoma.cl (Á.A.-D.); 4 Motor Control Research Group, Faculty of Sport Sciences, University of Extremadura, 10003 Cáceres, Spain; jesusmorenas@unex.es

**Keywords:** chronic disease, knowledge of specific disease, quality of life, disability

## Abstract

Introduction: Fibromyalgia (FM) translates into a reduction in the quality of life of people who suffer from it, being a chronic disease of unknown etiology. One of the most widespread treatments includes the combination of patient education, along with other components. At the educational level, the Fibromyalgia Knowledge Questionnaire (FKQ) is a tool that assesses knowledge of fibromyalgia. Objective: To obtain the translation and cultural adaptation of the FKQ questionnaire into Spanish, as well as its readability, in addition to knowing the relationship between knowledge of the disease and the level of disability. Method: In phase one, a translation-back translation and an evaluation of the readability of the questionnaire was carried out from INFLESZ, while in phase two, the questionnaire was passed to women with FM to detect their knowledge of the disease. A total of 49 women participated, with a mean age of 54.48 years. Results: The Spanish version of the FKQ questionnaire was rated by the participants in all its items as “clear and understandable”. The readability obtained by the questionnaire was similar to its original version, with both totals being in the “normal” range, following the INFLESZ ranges. Regarding the patients’ knowledge about FM, the component in which the highest score was obtained was physical activity (80% correct), while the one that obtained the worst score was knowledge about medication (50% correct). In addition, an inverse correlation was obtained between the FKQ and the FIQ (Fibromyalgia Impact Questionnaire) (r = −0.438; *p* < 0.01). Conclusions: The FKQ has been translated and culturally adapted, obtaining a correct understanding by the participants, as well as a degree of readability similar to the original questionnaire. Furthermore, it was obtained that, the lower the level of knowledge of the sick person, the greater the disability.

## 1. Introduction

Currently, fibromyalgia (FM), recognized as a disease in the Copenhagen Declaration by the World Health Organization in 1992 [1], results in a reduction in the quality of life of people who suffer from it [2], being a chronic disease of unknown etiology that involves generalized pain and other associated symptoms such as intense fatigue, non-restorative sleep, as well as numerous somatic and cognitive FM symptoms [3]. FM affects an average of 2.10% of the world’s population; 2.31% of the European population [4]; and, according to the Spanish Society of Rheumatology, between 2% and 6% of the Spanish population, with a female/male 20/1 ratio [5].

Nowadays, the origin or cause of FM is unknown, however, there are various hypotheses about its appearance [6]: genetic/hereditary factors, dysfunction of the hypothalamic-pituitary-adrenal axis, immunological changes, autoantibody production, autonomic nervous system dysfunction, environmental factors, microbiological origin (virus or mycoplasma), stress, or trauma. In general, and what almost all researchers agree on, is that FM is a brain origin disease, as there are abnormalities in the perception of pain and neurochemicals at the brain level; that is, high levels of substance P have been detected [7,8], as well as lower levels of growth hormone (GH) and cortisol. These deficits would cause an increase in hyperprolactinemia, which in turn would reduce serotonin levels, a well-known pain inhibitor. Therefore, by presenting serotonin deficits, FM patients have a greater sensitivity to pain, being more present in their lives [9].

Although no treatments have been found thus far that make the symptoms that characterize it disappear, we can carry out some treatments to calm them. The most effective treatments for this pathology are based on multidisciplinary treatments [10,11], that is, a combination of drug intake, physical activity, and psychological treatment [12] or a combination of patient education, pain coping strategies, and aerobic exercise [13].

In relation to patient education from the point of view of multidisciplinary treatment, we can find some previous studies that report that this education associated with other types of treatments such as physical activity would provide very beneficial results [14,15,16]. More specifically, health education programs could modify the perception of quality of life and improve pain [17], in addition to improving health status and inhibiting endogenous pain in the long term [18].

Moretti, et al. [19] in their research on FM knowledge highlighted that an educational process should be carried out to inform the patient about the disease, symptoms and physical exercises, medication, treatment, and other aspects such as cognitive skills, thus it is necessary to create and validate tools that allow quantitative evaluation of this knowledge. For this, the Fibromyalgia Knowledge Questionnaire (FKQ) [20] was created, which is composed of 18 items, divided into 4 domains (general knowledge about the disease, knowledge about treatment, medication and possible side effects, knowledge about physical activity, and knowledge about day-to-day activities in relation to the energy used or the best way to save it). However, this questionnaire was in Portuguese, so the need arises for its translation and cultural adaptation to Spanish. This translation and adaptation is necessary to ensure that the results are not contaminated owing to some misunderstanding or misinterpretation by the patient [21,22] because, on occasion, a literal translation from a foreign language can lead to conceptual errors or unfamiliar references in the subject’s language. In the same way, an adaptation that is too far from the original resource can introduce new variables or condition pre-existing variables, giving them a different meaning from the original one. That is why it is essential to verify the validity of the adapted and translated resource. Likewise, the translation and adaptation of this type of tools, methodologically accepted in other countries, can facilitate a fluid exchange of information and knowledge in the international scientific community [23]. Thus, the objective of this study was to obtain the translation and cultural adaptation of the FKQ questionnaire into Spanish, as well as its readability. In addition, a secondary objective was established as knowing the relationship between knowledge of the disease and their level of disability.

## 2. Materials and Methods

### 2.1. Ethical Approval

Ethical approval was provided by the Bioethics and Biosafety Committee of the University of Extremadura (approval number: 51/2013).

### 2.2. Procedures

**Figure 1:**
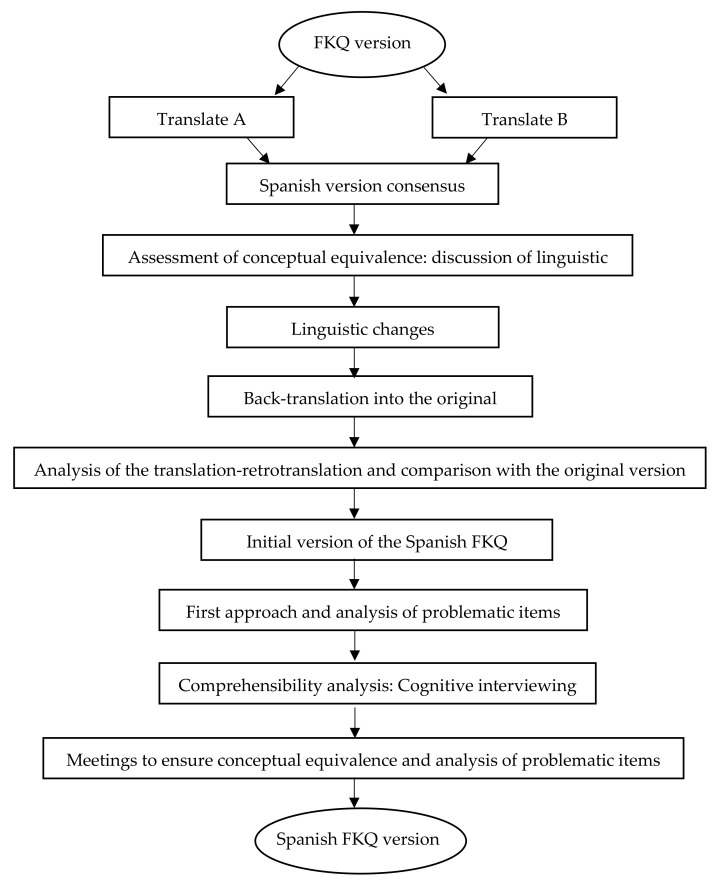
Translation and cultural adaptation procedure of the Spanish version of the Fibromyalgia Knowledge Questionnaire (FKQ).

The procedure of the present study was the methodology used in a translation-back-translation process and evaluation of the readability of the questionnaire (Figure 1). For this, the direct and inverse translation methodology was applied, as in previous studies [24], such as the translation and adaptation of the EQ-5D-Y [25], and the Questionnaire of Detection of Mild Cognitive Disorder [26].

**Phase 1:** Obtaining the Spanish version of the FKQ questionnaire. Translation and cultural adaptation.

It began by translating the FKQ questionnaire by two Spanish translators with command of the original language (Portuguese), where they rated from 0 to 10 the level of difficulty they had when translating each question, where 0 represents no difficulty and 10 very difficult. After the independent translation process by the two translators of the questionnaire, a consensus meeting was convened to obtain a single translation and cultural adaptation of the questionnaire, carrying out the discussion of linguistic differences to obtain the best possible readability of those affected, obtaining in this way the initial consensus of the FKQ.

Once the translation into Spanish was obtained, backtranslation was carried out, based on translating the questionnaire previously translated into Spanish by a translator with a Portuguese mother tongue with command of the Spanish language into the original language. Next, the original questionnaire version was compared (in Portuguese) with the back-translated questionnaire, making a comparison and finally obtaining the correctly translated questionnaire. The Spanish version was evaluated for comprehension in a sample of 15 healthy people between the ages of 30 and 60 years. Participants rated the questionnaire overall as clear and understandable. The conducted interviews were evaluated through three methods:

- Comprehension on an ordinal scale using a three-point scale: (1) clear and understandable, (2) difficult to understand, and (3) incomprehensible.
- Evaluation of comprehension on a numerical scale using a scale from 0 to 10, with 0 being very easy to understand and 10 very difficult to understand.
- Inquiry and paraphrase where the interviewees expressed with their words the perceived meaning of the items of the questionnaire.

In addition to analyzing the readability with the three previously exposed methods, the questionnaire was analyzed with INFLESZ [27], a program based on the legibility of written texts addressed to patients, being an indicator of quality assistance. Szigriszt made an adaptation of the Flesch RES Scale, which he called the “Perspicuity Level Scale”, to measure the difficulty of reading a text through the Flesch–Szigriszt index, defining the parameters as follows: less than 40 points, very difficult degree of readability; between 40 and 55 points, somewhat difficult degree of readability; between 55 and 65 points, normal; between 65 and 80 points, quite easy; and greater than 80 points, very easy.

**Phase 2:** Filling in the FKQ questionnaire.

Once the objectives of the study had been defined and the measurement instruments considered adequate to collect the relevant information had been selected, a dossier was prepared with them, adding data of interest such as weight, height, body mass index, years of accurate diagnosis, years of first symptoms, number of household members, household income, income/members, and number of trigger points.

Subsequently, the different associations were contacted to inform those affected about the study, they were summoned, and the objective of the study was explained to them, and they proceeded to answer the questionnaire. This was administered by an experienced interviewer.

Before completing the questionnaires, the practitioners were given a series of instructions based on the mechanics of filling in the questionnaires and they were informed about the main objective of the study.

### 2.3. Participants

The total sample of participants for the comprehensibility of the questionnaires was 15 women (5 for each following age range: between 30 and 40, 41 and 50, and 51 and 60, respectively) from the towns of Don Benito and Mérida, where their ability to understand the different items of the questionnaire was analysed.

For the second part of the study, in which the questionnaires were filled in, the total sample was 49 women aged 30 to 60, with a mean of 54.48 years. In this case, the participants were recruited from four associations of patients affected by FM syndrome (from the cities Mérida, Don Benito, Valencia de Alcántara, Alburquerque, Toledo, and Almansa).

The inclusion criteria were as follows: to be of legal age, to have a diagnosis of FM by a rheumatologist according to the American College of Rheumatology criteria [28], not to present any disease affecting the understanding of the test, and to sign the informed consent form.

### 2.4. Instruments and Measurement

During the present study, various instruments were used, more specifically, questionnaires, which are detailed below:

- Sociodemographic questionnaire: In which personal data such as date of birth, sex, number of members of the family unit, income level, studies carried out, occupation, if the patient receives non-pharmacological therapy, in addition to the year of initiation and diagnosis were collected, as well as symptoms, trigger points, current pain scale, and lastly weight and height.
- Europaliq: This questionnaire is made up of five items. The first one refers to the number of cigarettes he smokes, with five possible answers. The second to the frequency of alcoholic beverages, with seven possible answers. The third to the number of meals made per day, with four possible answers. The fourth to the hours of physical exercise with eight factors. Lastly, the fifth item composed of six factors referring to the distance walked daily.
- Fibromyalgia Impact Questionnaire (FIQ) [29]. The questionnaire is made up of 10 items. The first item with 10 factors valued from 0 to 3 with 0 = always and 3 = never. The second and third items are valued on a scale from 0 to 7, with the number of days a week and all the remaining items being valued on a scale from 0 to 10, with 0 being the least discomfort and 10 being the maximum discomfort. Participants completed the Spanish version (Spearman correlation = 0.76; *p* < 0.001).
- Fibromyalgia Knowledge Questionnaire (FKQ): Questionnaire validated by Suda, Jennings, Bueno, and Natour [20]. This questionnaire on knowledge about FM consists of 18 items divided into 4 domains (general knowledge about the disease; knowledge about treatment, medication, and possible side effects; knowledge about physical activity; and knowledge about day-to-day activities in relation to energy used or how best to save energy), on how much the subject knows about FM and how it can affect her life. For each item, one or more answers can be chosen. The total score of the questionnaire reached 26 points, where the higher score corresponds to higher knowledge (Cronbach’s alpha > 0.69).

### 2.5. Stadistical Analysis

The information collected was transferred to an anonymized database designed specifically for the study. The IBM SPSS Statistics software (Version 21, IBM SPSS, Chicago, IL, USA) was used.

First, the normality of each of the study variables was analysed using the Shapiro–Wilk Test and, once it was verified that the data did not follow a normal distribution, the Spearman correlation coefficient was calculated between the FKQ, FIQR questionnaires, and FIQ. The type of correlation was defined following Cohen [30]: 0.30 to 0.59, moderate; 0.60 to 0.79, high; and ≥0.80, excellent.

## 3. Results

### 3.1. Phase 1

After carrying out the comprehensibility questionnaires to the 15 participants who were part of this phase, problems were obtained in several items. In item 4c, which states “Perform laboratory tests such as blood counts and hormone levels”, “blood analysis” was specified in parentheses for better understanding; in item 13a, “Hydrotherapy”, “use of water as a therapeutic agent” was specified in parentheses; in item 13 c, “Ultrasound” was specified with the clarification “therapy with acoustic or sound waves”; finally, item 17 a, “In FM, the use of orthoses in the painful joint is always indicated” was clarified with the exemplification “such as splints or other technical aids”.

Therefore, once these changes were made, the FKQ was understood in all its items, without difficulty, with the lowest possible score prevailing on both the ordinal and numerical scales.

An analysis of objective readability was also carried out through the program called INFLESZ [27]. The readability of the adapted Spanish version was very similar to that of the questionnaire in its original version, finding the total of both at the “normal” level following the INFLESZ score ranges [27]. However, items 4, 6, 7, 8, 10, 13 and 18 obtained scores below 59 in both languages (Table 1), which is why they are considered to be difficult to read.

### 3.2. Phase 2

In the second phase, after detecting, correcting the terms with difficulty of under-standing and obtained the final version of Fibromyalgia Knowledge Questionnaire (Spanish version) (Please see Supplementary Materials), a statistical analysis was carried out to obtain the results of the questionnaires.

Table 2 shows the mean characterization of the patients who participated in the study seeing high perceived pain and number of trigger points.

All of the patients are women with primary education or school graduates, and most of them are housewives and unemployed persons (Table 3).

Regarding their lifestyle, they do not smoke or drink alcoholic beverages, they eat at least one hot meal every day, and they are sedentary, as can be seen in Table 4, where the characteristics of lifestyle habits are expressed.

Regarding the correct questions answered in the FKQ, Table 5, the existence of greater general knowledge (91.83%) and knowledge about exercise and energy (85.71%) that the participants have about the disease stands out. In contrast, great difficulty in understanding the diagnosis of FM was detected (2.04%).

Table 6 shows the median and interquartile range of the different components of the FKQ and the total score. In it, it can be seen that the component in which the highest score was obtained was physical activity (80% correct), while the one that obtained the worst score was knowledge about medication (50% correct).

Furthermore, an inverse relationship between the FIQ and FKQ was observed (r: −0.438; *p* < 0.001). Thus, a higher score in the FIQ (from 0–100) is related to a lower score in FKQ (0–26), meaning that less knowledge leads to greater disability.

## 4. Discussion

The main finding of this study was to obtain an adequate translation into Spanish, as well as cultural adaptation and readability of the FKQ questionnaire originating in the Portuguese language, to be applied in Spanish women with FM.

For the translation and cultural adaptation of the FKQ to its Spanish version, we followed the direct and inverse translation methodology, as previous studies reported [24,25,26]. This version reported similar comprehensibility results to other studies in which this aspect has been evaluated in people with FM [31]. Specifically, FKQ has been understood in all its items, because, for higher comprehensibility, items 4c, 13a, 13c, and 17a were specified in parentheses or with an exemplification for better understanding. The readability of the adapted Spanish version was very similar to that of the questionnaire in its original version, with the total of both at the “normal” level according to the INFLESZ score ranges [27]. Specifically, items (4, 6, 7, 8, and 13) showed a “very difficult” or “somewhat difficult” readability; these items also had similar readability in the original items [20]. In addition, other studies that evaluated readability on FM information have reported similar readability [32,33], classifying some information as “slightly difficult to read” [32]. In contrast, in the first phase of the study, they presented good comprehensibility. Therefore, they could be understandable, but not very readable, so that an alternative wording could be valuable for them.

Regarding the knowledge of the participants, similarities were observed in the mean between the scores obtained by the participants in this study and the participants who took part in the validation study of the original FKQ questionnaire (Table 7), finding in our study a level of greater knowledge, with a difference of 1.28 points in the total questionnaire [34].

In this regard, some studies attempted to address the knowledge of specialists about FM. Ortiz, et al. [34] concluded that knowledge in diagnosis and treatment of FM in primary care physicians in Chiclayo was deficient. More specifically, concerning the scores obtained about knowledge in the participants of this study (Table 7), it can be seen that one of the lowest scores is found in the medication domain; this could be justified by the lack of guidance from their physicians on the suitability of the different treatments or drugs, because, as highlighted in several studies such as that of Blotman, et al. [35], 20% of the physicians participating in their study took nonsteroidal anti-inflammatory drugs as appropriate medication. In this line, Kianmehr, et al. [36], pointed out that 53.2% of the GPs participating in their study had low or very low levels of knowledge about the treatment of FM, and more specifically, 52.1% of them also marked the use of nonsteroidal anti-inflammatory drugs.

Several studies have documented that exercise interventions improve fibromyalgia pain [37,38]. Rahman, et al. [39] observed that exercise therapy could be even better than pharmacological interventions as a treatment for fibromyalgia. However, it has been shown FM patients are less physically active than healthy women [40]. In this sense, education about physical activity can be key, as Burton, et al. [41] reported that initiation of physical activity was significantly related to the patient’s belief in the importance of physical activity, in addition to age and current health status. In addition, they established as predictors of maintenance (more than 4 years) of physical activity the education and belief that exercise contributed to good health, among other aspects [41]. Therefore, education in this sense and thus the possible practice could be translated into an improvement in the health of patients.

Following up with the benefits that could be caused by improving the patient’s knowledge of the disease, the second finding of this study was the existence of a relationship between the level of knowledge and disability, being inversely proportional such that, the less knowledge about FM, the greater the disability. In this line, various studies state the knowledge of patients about the disease contributes to its control [42,43]. The evaluation of an FM education programme has been little studied, so further studies would be relevant to determine the level of influence or weight that knowledge of the disease could have in such multidisciplinary programmes. Because, as various studies show [43,44], an education program about the disease could both decrease the impact of FM and improve HRQoL levels.

In this line, possible improvements both at the level of the patient and the symptomatology, as well as at the level of the health service, if this would allow cost reduction, could be beneficial for patients as already being in other diseases such as diabetes [45,46,47] or arthritis [48,49], where there are many educational programmes aimed at different populations.

Regarding the limitations of the study, it should be mentioned that the sample was relatively small and it was only applicable to women with FM, as the study has not been carried out with men. It can also be highlighted that the results would not apply to women over 65 years of age without studies, as they were not included in this study either.

The strength of this study is that the results provided preliminary evidence of the potential utility of the FQK in Spanish for assessing FM knowledge of patients with FM. In this sense, the assessment of patients’ knowledge can help to check their level, and from epidemiological studies, help managers to adequately carry out how they should guide public policy education programs in FM.

## 5. Conclusions

The results of the present study allow us to conclude that the FKQ questionnaire has been translated and culturally adapted, obtaining a correct understanding by the participants, as well as a degree of readability similar to the original questionnaire. In addition, an inverse relationship was obtained between the Fibromyalgia Impact Questionnaire and the FKQ, that is, the less the knowledge of the patient, the greater the disability. Thus, the most important future line that is being proposed to us is to analyze its reliability and validity with a representative sample of both rural and urban population, as well as the possible validation in another type of population such as the family environment of the patients or even the specialists themselves.

## Figures and Tables

**Table 1:** FKQ readability in Spanish and original language from INFLESZ.

Item	INFLESZ Score	
	Spanish Version	Original Version
1	59.67	63.96
2	66.85	49.48
3	91.07	69.83
4	51.18	56.92
5	67.75	66.26
6	25.18	31.00
7	48.36	57.70
8	38.05	40.89
9	70.84	68.41
10	46.54	59.22
11	67.39	68.47
12	54.52	67.56
13	47.08	46.13
14	84.42	73.79
15	74.40	68.89
16	83.55	79.17
17	71.04	72.43
18	59.53	55.00
**Total**	63.92	63.45

Percentages <44 level, “very difficult”; from 45 to 59, “somewhat difficult”; 60 to 69, “normal”; 70 to 84, “pretty easy”; ≥85, “very easy”.

**Table 2:** Sociodemographic characteristics of FM patients.

	N	Mean (SD)
Age (years)	47	54.48 (12.56)
Weight (Kg)	43	73.86 (18.19)
Height (Cm)	45	157.28 (12.79)
Body mass index (Kg/m^2^)	41	48.38 (24.38)
Years of accurate diagnosis (years)	43	9.11 (6.51)
Years of appearance of the first symptoms (years)	44	17.95 (10.29)
Number of members of the family unit	49	3.02 (1.31)
Family unit income (Euros)	40	1018.62 (464.05)
Income/members (Euros)	40	401.62 (220.21)
Number of trigger points	37	16.59 (2.11)
Visual analogue scale of perceived pain intensity *	48	74.33 (21.57)

* On the visual analog scale, 0 is the absence of pain and 100 is the maximum pain.

**Table 3:** Characteristics of the patients participating in the study.

	N = 49
**Level of Studies**	**N (%)**
No studies, but can read and write	14 (28.6)
Primary studies or school graduate	25 (51)
Vocational or secondary training	8 (16.3)
First and second cycle or degree	1 (2)
Other	1 (2)
**Employment Situation**	**N (%)**
Self-employed	1 (2)
Works for someone else	1 (2)
Official	2 (4.1))
Unemployed	12 (24.5)
Retired	9 (18.4)
Housewife	23 (46.9)
Student	1 (2)

**Table 4:** Characteristics of lifestyle habits.

How many cigarettes, cigars, or pipes do you smoke daily?	N (%)
From 15 to 24	4 (8.2)
From 5 to 14	9 (18.4)
From 1 to 4	5 (10.2)
Don’t smoke	30 (61.2)
Lost	1(2)
How often do you drink alcoholic beverages over two bottles of beer or two glasses of liquor?	N (%)
Daily	2 (4.1)
2 or more times a week	1 (2)
Once a week	3 (6.1)
2 or 3 times a month	4 (8.2)
Less than once a month	5 (10.2)
I never take more than the amount indicated	2 (4.1)
I never drink alcoholic beverages	30 (61.2)
How many hot meals do you eat a day?	N (%)
From time to time I take some	3 (6.1)
At least one every day	30 (61.2)
At least two every day	16 (32.7)
How many hours a week do you dedicate to physical exercise?	N (%)
None	5 (10.2)
Less than 1 h	10 (20.4)
Between 1 and 2 h	12 (24.5)
Between 3 and 4 h	12 (24.5)
Between 5 and 8 h	7 (14.3)
Between 9 and 14 h	2 (4.1)
Between 15 and 21 h	1 (2)
Approximately, indicate the distance you walk daily	N (%)
I don’t walk/very little	13 (26.5)
Less than a km	3 (6.1)
Between 1 and 2 km	20 (4.8)
Between 3 and 5 km	11 (22.4)
Between 6 and 9 km	1 (2)
More than 9 km	1 (2)

**Table 5:** Frequency of correct answers about the FKQ.

Item	Question	(%)
1	Please check two correct choices about the cause of fibromyalgia:	22.44
2	What are the main symptoms of fibromyalgia? Check two correct options:	59.18
3	In addition to the symptoms mentioned in the previous question, choose another (just one) that can occur in fibromyalgia:	91.83
4	What is necessary to confirm the diagnosis of fibromyalgia? Check two correct options:	2.04
5	Over time, what can happen to the patient due to fibromyalgia? Check two correct options:	55.10
6	Indicate a correct option about the most indicated medications in the treatment of fibromyalgia:	77.55
7	Choose a correct option in terms of prescription drugs for the treatment of fibromyalgia:	40.81
8	What are the best fibromyalgia treatment combinations? Check two correct options:	24.48
9	What are the most common side effects that the medications used in fibromyalgia can cause? Check two correct options:	36.73
10	Check one correct option for physical activity in fibromyalgia treatment:	65.30
11	What is the importance of exercises for those who have fibromyalgia? Check one correct option:	42.85
12	What is the best way for a fibromyalgia patient to exercise? Check one correct option:	85.71
13	Choose the two best methods that can be used in the rehabilitation of the fibromyalgia patient:	57.14
14	What is the best way to conserve your energy? Choose one correct option:	85.71
15	What are the other good ways to keep your vitality? Check two correct options:	81.63
16	What is the best way to protect the joints? Check one correct option:	69.38
17	Please mark one correct option as to the best form of joint protection:	6.12
18	Please mark one correct option about fibromyalgia:	81.63

Questionnaire carried out on 49 participants (100%).

**Table 6:** Total score and by component of the FKQ.

	FKQT	FKQG	FKQM	FKQAF	FKQE
Median (IR)	17(5)	6(3)	3(1)	4(1.5)	4(1)
Hit percentage	65.3%	66.6%	50%	80%	66.6%

FKQT: Fibromyalgia Knowledge Questionnaire total, with 0 points being no knowledge and 26 maximum knowledge; FKQG: general knowledge about fibromyalgia (question 1–5) = 9 points; FKQM: knowledge about medication (question 6–9) = 6 points; FKQAF: knowledge about physical activity (question 10–13) = 5 points; FKQE: knowledge about energy (question 14–18) = 6 points; data are expressed as median and interquartile range (IR).

**Table 7:** Average scores obtained in each dimension of the FKQ in Spanish and in the original study.

	FKQT	FKQG	FKQM	FKQAF	FKQE
	Mean (SD)	Mean (SD)	Mean (SD)	Mean (SD)	Mean (SD)
Spanish	16.48(3.64)	5.59(1.56)	3.36(1.21)	3.30(1.51)	4.22(0.82)
Original [20]	15.20(4.20)	6.20(2.10)	2.80(1.36)	3.40(1.14)	2.80(1.10)

SD: standard deviation; FKQT: Fibromyalgia Knowledge Questionnaire total, with 0 points being no knowledge and 26 maximum knowledge; FKQG: general knowledge about fibromyalgia (question 1–5) = 9 points; FKQM: knowledge about medication (question 6–9) = 6 points; FKQAF: knowledge about physical activity (question 10–13) = 5 points; FKQE: knowledge about energy (question 14–18) = 6 points.

## Data Availability

The datasets used during the current study are available from the corresponding author on reasonable request.

## Supplementary Materials

The following are available online at https://www.mdpi.com/article/10.3390/ijerph18147678/s1, Fibromyalgia Knowledge Questionnaire (Spanish version).

## References

[1] Csillag C. (1992). Fibromyalgia: The Copenhagen declaration. Lancet.

[2] Mease P.J., Arnold L.M., Crofford L.J., Williams D.A., Russell I.J., Humphrey L., Abetz L., Martin S.A. (2008). Identifying the clinical domains of fibromyalgia: Contributions from clinician and patient Delphi exercises. Arthritis Rheum..

[3] Wolfe F., Smythe H.A., Yunus M.B., Bennett R.M., Bombardier C., Goldenberg D.L., Tugwell P., Campbell S.M., Abeles M., Clark P. (1990). The American College of Rheumatology 1990 Criteria for the Classification of Fibromyalgia. Report of the Multicenter Criteria Committee. Arthritis Rheum..

[4] Cabo-Meseguer A., Cerdá-Olmedo G., Trillo-Mata J.L. (2017). Fibromialgia: Prevalencia, perfiles epidemiológicos y costes económicos. Med. Clínica.

[5] Ubago Linares M.d.C., Ruiz Pérez I., Bermejo Pérez M.J., Labry Lima A.O.d., Plazaola Castaño J. (2005). Características clínicas y psicosociales de personas con fibromialgia: Repercusión del diagnóstico sobre sus actividades. Rev. Española Salud Pública.

[6] Helfenstein M., Goldenfum M.A., Siena C.A. (2012). Fibromyalgia: Clinical and occupational aspects. Rev. Assoc. Med. Bras..

[7] Russell I.J. (1989). Neurohormonal aspects of fibromyalgia syndrome. Rheum. Dis. Clin. N. Am..

[8] Russell I.J., Orr M.D., Littman B., Vipraio G.A., Alboukrek D., Michalek J.E., Lopez Y., MacKillip F. (1994). Elevated cerebrospinal fluid levels of substance P in patients with the fibromyalgia syndrome. Arthritis Rheum..

[9] Neeck G., Riedel W. (1994). Neuromediator and hormonal perturbations in fibromyalgia syndrome: Results of chronic stress?. Bailliere’s Clin. Rheumatol..

[10] Cruz A.C., i Mata X.T., i Gassol A.A., Gabaroi D.C., Vilarrasa R., Miyar M.V., Muñoz-Gómez J. (2001). Eficacia del tratamiento multidisciplinario del dolor crónico incapacitante del aparato locomotor. Med. Clínica.

[11] Navarro E.A. (2017). Efectos de un Programa Multidisciplinar Sobre el Estado de Salud y Procesos Psicológicos en Personas con Fibromialgia.

[12] Villanueva V.L., Valía J.C., Cerdá G., Monsalve V., Bayona M.J., Andrés J.D. (2004). Fibromialgia: Diagnóstico y tratamiento. El estado de la cuestión. Rev. Soc. Española Dolor.

[13] Carbonell-Baeza A., Aparicio V., Chillón P., Femia P., Delgado-Fernandez M., Ruiz J. (2011). Effectiveness of multidisciplinary therapy on symptomatology and quality of life in women with fibromyalgia. Clin. Exp. Rheumatol. Incl. Suppl..

[14] Rooks D.S., Gautam S., Romeling M., Cross M.L., Stratigakis D., Evans B., Goldenberg D.L., Iversen M.D., Katz J.N. (2007). Group exercise, education, and combination self-management in women with fibromyalgia: A randomized trial. Arch. Intern. Med..

[15] Hassett A.L., Gevirtz R.N. (2009). Nonpharmacologic treatment for fibromyalgia: Patient education, cognitive-behavioral therapy, relaxation techniques, and complementary and alternative medicine. Rheum. Dis. Clin..

[16] Novo J.P., Pereira A.E., García A.R., Martín R.S., Méndez B.G. (2015). Guía para la rehabilitación de la fibromialgia. Rev. Cuba. Reumatol..

[17] Romero E.B., Moya N.S., Esteve M.V., Valer S.V. (2002). Estudio de la calidad de vida en pacientes con fibromialgia: Impacto de un programa de educación sanitaria. Atención Primaria.

[18] Van Oosterwijck J., Meeus M., Paul L., de Schryver M., Pascal A., Lambrecht L., Nijs J. (2013). Pain physiology education improves health status and endogenous pain inhibition in fibromyalgia: A double-blind randomized controlled trial. Clin. J. Pain.

[19] Moretti F.A., Heymann R.E., Marvulle V., Pollak D.F., Riera R. (2011). Assessing knowledge on fibromyalgia among Internet users. Rev. Bras. Reum..

[20] Suda A.L., Jennings F., Bueno V.C., Natour J. (2012). Development and validation of fibromyalgia knowledge questionnaire: Fkq. Rheumatol. Int..

[21] Wlodyka-Demaille S., Poiraudeau S., Catanzariti J.-F., Rannou F., Fermanian J., Revel M. (2002). French translation and validation of 3 functional disability scales for neck pain. Arch. Phys. Med. Rehabil..

[22] Guillemin F., Bombardier C., Beaton D. (1993). Cross-cultural adaptation of health-related quality of life measures: Literature review and proposed guidelines. J. Clin. Epidemiol..

[23] Hutchinson A., Bentzen N., König-Zahn C. (1997). Cross-Cultural Health Outcome Assessment: A User’s Guide.

[24] Lohr K.N. (2002). Assessing health status and quality-of-life instruments: Attributes and review criteria. Qual. Life Res..

[25] Gusi N., Badia X., Herdman M., Olivares P.R. (2008). Translation and cultural adaptation of the Spanish version of EQ-5D-Y questionnaire for children and adolescents. Aten. Primaria.

[26] Ojeda B., Salazar A., Duenas M., Failde I. (2011). Translation and adjustment into Spanish language of the screening tool for mild cognitive impairment. Med. Clin..

[27] Barrio-Cantalejo I.M., Simón-Lorda P., Melguizo M., Escalona I., Marijuán M.I., Hernando P. (2008). Validación de la Escala INFLESZ para evaluar la legibilidad de los textos dirigidos a pacientes. An. Sist. Sanit. Navar..

[28] Wolfe F., Clauw D.J., Fitzcharles M.A., Goldenberg D.L., Katz R.S., Mease P., Russell A.S., Russell I.J., Winfield J.B., Yunus M.B. (2010). The American College of Rheumatology preliminary diagnostic criteria for fibromyalgia and measurement of symptom severity. Arthritis Care Res..

[29] Esteve-Vives J., Redondo J.R., Salvat M.I.S., de Gracia Blanco M., de Miquele C.A. (2007). Proposal for a consensus version of the Fibromyalgia Impact Questionnaire (FIQ) for the Spanish population. Reumatol. Clínica (Engl. Ed.).

[30] Cohen J. (1988). Statistical power analysis for the behavioral sciences: Jacob Cohen. J. Am. Stat. Assoc..

[31] Vaidya B., Nakarmi S., Bhochhibhoya M., Joshi R. (2020). Translation, validation and cross-cultural adaptation of the Revised Fibromyalgia Impact Questionnaire (FIQR) in Nepali language. Int. J. Rheum. Dis..

[32] Cheneguin A.A., Salvat I.S., Barrero H.R., Lacomba M.T. (2020). How good is online information on fibromyalgia? An analysis of quality and readability of websites on fibromyalgia in Spanish. BMJ Open.

[33] Castillo-Ortiz J.D., de Jesus Valdivia-Nuno J., Ramirez-Gomez A., Garagarza-Mariscal H., Gallegos-Rios C., Flores-Hernandez G., Hernandez-Sanchez L., Brambila-Barba V., Castaneda-Sanchez J.J., Barajas-Ochoa Z. (2017). Readability, relevance and quality of the information in Spanish on the web for patients with rheumatoid arthritis. Reumatol. Clínica (Engl. Ed.).

[34] Ortiz F.E.A., de la Cruz V.A.C., Jiménez F.E.L. (2017). Conocimientos de fibromialgia en médicos de atención primaria de la provincia de Chiclayo-Perú, 2016. Reumatol. Clínica.

[35] Blotman F., Thomas E., Myon E., Andre E., Caubere J., Taieb C. (2005). Awareness and knowledge of fibromyalgia among French rheumatologists and general practitioners. Clin. Exp. Rheumatol..

[36] Kianmehr N., Haghighi A., Bidari A., Sharafian Ardekani Y., Karimi M.A. (2017). Are general practitioners well informed about fibromyalgia?. Int. J. Rheum. Dis..

[37] Segura-Jiménez V., Carbonell-Baeza A., Aparicio V., Samos B., Femia P., Ruiz J., Delgado-Fernández M. (2013). A warm water pool-based exercise program decreases immediate pain in female fibromyalgia patients: Uncontrolled clinical trial. Int. J. Sports Med..

[38] Segura-Jiménez V., Romero-Zurita A., Carbonell-Baeza A., Aparicio V., Ruiz J., Delgado-Fernández M. (2014). Effectiveness of tai-chi for decreasing acute pain in fibromyalgia patients. Int. J. Sports Med..

[39] Rahman A., Underwood M., Carnes D. (2014). Fibromyalgia. BMJ.

[40] Mcloughlin M.J., Colbert L.H., Stegner A.J., Cook D.B. (2011). Are women with fibromyalgia less physically active than healthy women?. Med. Sci. Sports Exerc..

[41] Burton L.C., Shapiro S., German P.S. (1999). Determinants of physical activity initiation and maintenance among community-dwelling older persons. Prev. Med..

[42] Koca T.T., Tugan C.B., Koçyiğit B.F., Nacitarhan V. (2019). Fibromyalgia awareness in women aged between 18 and 75 years: A current view to fibromyalgia. J. Public Health.

[43] Rodríguez M.d.M.L. Education in fibromyalgia. A systematic review. Proceedings of the Jornadas Internacionales de Investigación en Educación y Salud: Educación y Salud en una Sociedad Global.

[44] Oh T.H., Stueve M.H., Hoskin T.L., Luedtke C.A., Vincent A., Moder K.G., Thompson J.M. (2010). Brief interdisciplinary treatment program for fibromyalgia: Six to twelve months outcome. Am. J. Phys. Med. Rehabil..

[45] Pérez Gutiérrez C. (2017). Educación para la Salud en la Mujer con Diabetes Gestaciona.

[46] Canché-Aguilar D.L., Zapata-Vázquez R.E., Rubio-Zapata H.A., Cámara-Vallejos R.M. (2019). Efecto de una intervención educativa sobre el estilo de vida, el control glucémico y el conocimiento de la enfermedad, en personas con diabetes mellitus tipo 2, Bokobá, Yucatán. Rev. Biomédica.

[47] Modrego B.A. (2019). Programa de Salud Dirigido a Adolescentes con Diabetes Mellitus Tipo I.

[48] Esquivel García J.A., Gale Ojeda J.V., Rodríguez Florido F., Rodríguez Galindo D.G., Sánchez Ramos K.J. (2018). Evaluación de un Programa Educativo en Salud para Pacientes con Artritis Reumatoide en Bogotá.

[49] Lopez-Olivo M.A., Ingleshwar A., Volk R.J., Jibaja-Weiss M., Barbo A., Saag K., Leong A., Suarez-Almazor M.E. (2018). Development and pilot testing of multimedia patient education tools for patients with knee osteoarthritis, osteoporosis, and rheumatoid arthritis. Arthritis Care Res..

