# Supplementary material for: Spanish Translation and Cultural Adaptation of the Fibromyalgia Knowledge Questionnaire
Source: Int J Environ Res Public Health. 2021 Jul 19;18(14):7678. doi: 10.3390/ijerph18147678 (PMC8305758; doi:10.3390/ijerph18147678)
Supplement: Supplementary file 1 [file ijerph-18-07678-s001.zip › ijerph-1267369-supplementary.pdf]

Fibromyalgia Knowledge Questionnaire (FKQ)- Spanish version

1.Señale **dos** opciones correctas sobre la causa de la fibromialgia:

a. El exceso de trabajo puede causar fibromialgia.
b. Los traumas físicos pueden desencadenar fibromialgia.
c. La causa de la fibromialgia es desconocida.
d. La fibromialgia puede estar provocada por el frío.
e. Existen varios factores asociados a la causa de la fibromialgia.
f. No lo sé.

2. ¿Cuáles son los principales síntomas de la fibromialgia? Marque **dos** opciones correctas:

a. Deformidades en las articulaciones.
b. Sueño no reparador.
c. Inflamación de las articulaciones (artritis).
d. Caída del cabello.
e. Dolor generalizado.
f. No lo sé.

3. Además de los síntomas mencionados en la pregunta anterior, elija otro (solo **uno)** que puede presentarse en la fibromialgia:

a. Vértigo.
b. Fiebre.
c. Alergia.
d. Cansancio o fatiga.
e. No lo sé.

4. ¿Qué es necesario para confirmar el diagnóstico de la fibromialgia? Marque **dos** opciones correctas:

a. Es importante realizar una consulta médica, con el historial y un examen físico del paciente.
b. Realizar pruebas sofisticadas como resonancia magnética y tomografía (TAC).
c. Realizar pruebas de laboratorio como hemogramas y niveles hormonales (análisis de sangre).
d. Siempre es necesario pedir algún tipo de prueba.
e. A veces es necesario pedir pruebas para excluir otras dolencias de acuerdo con la valoración del médico.
f. No lo sé.

5. Con el paso del tiempo ¿qué puede sucederle al paciente debido a la fibromialgia? Marque **dos** opciones correctas:

a. Puede necesitar una silla de ruedas y llegar a morir.
b. Puede mejorar a través de la medicación correcta y de ejercicios físicos específicos.
c. Puede tener dificultades, pero podrá trabajar.
d. Puede que necesite cirugía.
e. No lo sé.

6. Señale **una** opción correcta sobre los medicamentos más indicados en el tratamiento de la fibromialgia:

a. Analgésicos y antidepresivos.
b. Diuréticos y antiinflamatorios.
c. Relajantes musculares y corticoesteroides (corticoides para combatir el estrés, inflamación…).
d. Vitaminas e infusiones.
e. Cremas y geles de masaje.
f. No lo sé.

7. Elija **una** opción correcta en cuanto a los fármacos recetados para el tratamiento de la fibromialgia:

a. Sólo el uso de los fármacos cura la enfermedad.
b. Para el tratamiento se utilizan antidepresivos en dosis bajas.
c. Los antiinflamatorios, además de ser muy importantes, son los únicos que tratan la fibromialgia.
d. Todos los fármacos utilizados para la fibromialgia causan dependencia.
e. No lo sé.

8. ¿Cuáles son las mejores combinaciones de tratamiento para la fibromialgia? Señale **dos** opciones correctas:

a. Internar al paciente con fibromialgia y realizarle acupuntura.
b. Ejercicios físicos regulares y el uso de antiinflamatorios cuando sienta dolor.
c. Infiltración en la zona dolorida y hacer reposo.
d. Realizar actividad física regular y, si es necesario, terapia psicológica.
e. Realizar actividad física regular, como caminar o gimnasia en el agua, asociada a antidepresivos en dosis bajas.
f. No lo sé.

9. ¿Cuáles son los efectos secundarios más comunes que pueden causar los medicamentos utilizados en la fibromialgia? Señale **dos** opciones correctas:

a. Aumento de la presión arterial y vértigo.
b. Sequedad de la boca y somnolencia (sueño).
c. Aumento de peso y estreñimiento.
d. Problemas de piel y oculares.
e. No lo sé.

10. Marque **una** opción correcta en cuanto a la actividad física en el tratamiento de la fibromialgia:

a. El paciente con fibromialgia debe realizar ejercicios físicos intensos para mejorar.
b. La actividad física regular no mejora los síntomas de la fibromialgia.
c. No es necesario realizar ejercicios físicos si tiene una vida normalmente activa, como ir andando a la compra, llevar a los hijos al colegio e ir andando al trabajo.
d. Los ejercicios físicos pueden aumentar los niveles de serotonina (estado de ánimo), disminuir el dolor y mejorar el estado físico general.
e. No lo sé.

11. ¿Cuál es la importancia de los ejercicios para quien tiene fibromialgia? Marque **una** opción correcta:

a. Los ejercicios son importantes y sólo funcionan cuando causan dolor.
b. Cuando el paciente siente dolor, es mejor parar y descansar.
c. Los ejercicios físicos se deben realizar, al menos, tres veces por semana.
d. Para las personas que trabajan mucho, los ejercicios están contraindicados.
e. No lo sé.

12. ¿Cuál es la mejor manera en la que un paciente con fibromialgia puede hacer ejercicio físico? Señale **una** opción correcta:

a. Ejercicios de mantenimiento como caminatas regulares y gimnasia en el agua.
b. Hacer ejercicio el mismo día que haga la limpieza de su casa.
c. Subir y bajar escaleras.
d. Hacer ejercicios agotadores por su cuenta.
e. No lo sé.

13. Elija los **dos mejores métodos** que se pueden utilizar en la rehabilitación del paciente con fibromialgia:

a. Hidroterapia (utilización del agua como agente terapéutico).
b. Infrarrojos (luz roja).
c. Ultrasonidos (terapia con ondas acústicas o sonoras).
d. Estiramientos.
e. No lo sé.

14. ¿Cuál es la mejor manera de que conserve su energía? Elija **una** opción correcta:

a. Permanecer en reposo la mayor parte del tiempo.
b. Limpiar la casa de una sola vez para tener el resto del día libre.
c. Realizar pausas (intervalos de descanso) durante las actividades del día.
d. Realizar todas las actividades sin pedir ayuda.
e. No lo sé.

15. ¿Cuáles son las otras maneras correctas de que conserve su vitalidad? Marque **dos** opciones correctas:

a. No trabajar, descansar todo el día.
b. Planificar las actividades del día, descansando entre las tareas.
c. Tener un horario regular para dormir.
d. Dormir la mayor parte del tiempo para ahorrar energía.
e. No lo sé.

16. ¿Cuál es la mejor manera de proteger las articulaciones? Marque **una** opción correcta:

a. Empujar los objetos en vez de tirar de ellos.
b. Cargar las bolsas utilizando sólo una de las manos, poniendo todo el peso en un solo lado del cuerpo.
c. Doblar la cintura hacia delante para levantar objetos del suelo.
d. Sujetar el teléfono con la cabeza y el hombro.
e. No lo sé.

17. Marque **una** opción correcta en cuanto a la mejor forma de protección articular:
    a. En la fibromialgia, siempre se indica el uso de órtesis (como férulas u otras ayudas técnicas) en la articulación dolorida.
    b. Cargar las bolsas en el antebrazo (articulación grande) en vez de en las manos (articulación pequeña).
    c. Usar emplastes (vendajes) en las zonas doloridas.
    d. Colocar protecciones en los lugares con mayor dolor.
    e. No lo sé.

18. Señale **una** opción correcta sobre la fibromialgia:
    a. Los aspectos emocionales son los únicos responsables de la aparición de la enfermedad.
    b. Los factores estresantes no empeoran la enfermedad.
    c. Los pacientes con fibromialgia no pueden tener relaciones sexuales.
    d. La buena convivencia y el ambiente familiar no ayudan a la mejora de la fibromialgia.
    e. En algunos casos, puede ser necesaria la terapia psicológica además del tratamiento convencional.
    f. No lo sé.
